# *ABCB1 4036A*>*G* and *1236C*>*T* Polymorphisms Affect Plasma Efavirenz Levels in South African HIV/AIDS Patients

**DOI:** 10.3389/fgene.2012.00236

**Published:** 2012-11-05

**Authors:** Marelize Swart, Yuan Ren, Peter Smith, Collet Dandara

**Affiliations:** ^1^Division of Human Genetics, Faculty of Health Sciences, University of Cape TownObservatory, Cape Town, South Africa; ^2^Department of Clinical Pharmacology, Faculty of Health Sciences, University of Cape TownObservatory, Cape Town, South Africa

**Keywords:** *ABCB1*, efavirenz, HIV/AIDS, South Africa, pharmacogenetics

## Abstract

The *ABCB1* gene encodes P-glycoprotein, an ATP-dependent drug efflux pump, which is responsible for drug transport across extra- and intra-cellular membranes. The variability in the expression of *ABCB1* may contribute to variable plasma efavirenz concentration which results in variability in the levels of suppression of the human immunodeficiency syndrome virus (HIV). The aim of the study was to evaluate the role of polymorphisms in *ABCB1* gene on plasma efavirenz levels and treatment response in the form of change in viral load and CD-4 cell count in HIV/AIDS patients receiving efavirenz-containing highly active antiretroviral treatment regimens. Two hundred and eighty-two HIV-infected patients were recruited from Themba Lethu Clinic in Johannesburg and plasma efavirenz drug concentration levels were measured using LC-MS/MS. SNaPshot was used to genotype five known *ABCB1* single nucleotide polymorphisms (SNPs). Genotype-phenotype correlations were computed. The *ABCB1 4036A/G* and *4036G/G* genotypes were significantly associated with low plasma efavirenz concentrations (*P* = 0.0236), while the *ABCB1 1236C/T* and *1236T/T* genotypes were associated with high efavirenz concentrations (*P* = 0.0282). A haplotype *ABCB1 T-G-T-A* is reported that is associated with significantly increased plasma efavirenz levels. This is the first report on *61A*>*G*, *2677G*>*T/A*, and *4036A*>*G* SNPs in the South African population. *ABCB1* plays a role in determining the plasma concentrations of efavirenz and should be taken into account in future design of assays for genotype-based dosing of efavirenz-containing regimens.

## Introduction

Efavirenz provides the backbone to first-line highly active antiretroviral treatment (HAART) in South Africa. HAART effectively suppresses human immunodeficiency syndrome virus (HIV) replication in the majority of patients (Mocroft et al., 2003). Thus, many HIV-infected patients are now living longer compared to the pre-HAART period. However, long term antiretroviral (ARV) treatment has its own challenges such as drug–drug interactions and the development of adverse drug reactions (ADRs). Drug–drug interactions are a major problem in HIV/AIDS patients due to co-morbidities such as TB and malaria.

FDA-approved ARV drugs, including efavirenz, indinavir, nelfinavir, and ritonavir are affected by the activities of the multidrug transporter P-glycoprotein (P-gp), coded by the *ABCB1* gene. *ABCB1* forms part of the ATP-binding cassette gene family with about 50 members and is located on chromosome 7q21.12, spanning 209.6 kb, and containing 29 exons (Bodor et al., 2005). Genetic variation in the *ABCB1* gene is known to alter mRNA stability or splicing activity (Fung and Gottesman, 2009). The three most common single nucleotide polymorphisms (SNPs) in the protein coding region of *ABCB1* are *1236C*>*T* (*rs1128503*), *3435C*>*T* (*rs1045642*), and *2677G*>*T/A* (*rs2032582*), where multiple alleles have been reported. The *1236C*>*T* SNP occurring in exon 13, does not result in an amino acid change, but may affect *ABCB1* expression through codon usage (Gu et al., 2004). The allele frequency of *1236T* variant ranges from 10% among South Africans (Dandara et al., 2011) to 90% among Asians (Ambudkar et al., 1999). The *2677G*>*T/A* SNP results in a change from serine to alanine or threonine at residue 893, but the effect of the changes on protein function is uncertain. The *3435T* allele has been associated with reduced expression of P-gp, although it is synonymous (Meissner et al., 2004). Large inter-ethnic variability has been reported for the *3435C*>*T* SNP with the *ABCB1 3435C* variant being the most frequent at 83, 58, 57, and 11% among Africans (Kenyans and Ghanaians), Asians (Chinese), Caucasians, and Yoruba individuals, respectively (Ameyaw et al., 2001). The *ABCB1 3435T* variant has been linked with good immune recovery in HIV/AIDS individuals, while the presence of the *ABCB1 2677T* variant has been strongly associated with virological failure (Motsinger et al., 2006). A few studies have suggested associations between *ABCB1* gene polymorphisms and variability in plasma efavirenz concentrations (Fellay et al., 2002; Mukonzo et al., 2009), but all the studies lack adequate sample size. There are conflicting reports on the effects of these SNPs on efavirenz treatment response (Cascorbi et al., 2001; Fellay et al., 2002; Cascorbi, 2006). Replication studies are thus necessary to understand the contribution of *ABCB1* gene variants to plasma efavirenz levels. Dandara et al. (2011) showed that genetic variants in *ABCB1* are frequent in the South African population, and this study is a continuation further evaluating the clinical significance of these SNPs. Therefore, the aim of this study was to investigate the role of genetic polymorphisms in *ABCB1* on plasma efavirenz levels in HIV/AIDS patients in the South African population.

## Results

The mean age of the HIV/AIDS patients was 41.3 years, and more than 75% (*n* = 227) were female. Of the patients, 7 and 10% smoked and consumed alcohol. The clinical characteristic of the patients included viral load and CD-4 cell count (Table 1).

**Table 1:** **Clinical characteristics of the South African HIV/AIDS patients**.

Clinical characteristics	HIV/AIDS patients (*n* = 301)
Median HIV-RNA at baseline, copies/mL ± SD (range)	26917.71 ± 27133.50 (25–98400)
Median HIV-RNA at 6 months post-initiation of HAART, copies/mL ± SD (range)	1518.52 ± 9004.10 (0–75000)
Average CD-4 cell count at baseline, cells/μL ± SD (range)	136.09 ± 113.24 (2–605)
Average CD-4 cell count at 6 months post-initiation of HAART, cells/μL ± SD (range)	261.76 ± 137.68 (28–775)
ARV regimens	
3TC_TDF_EFV	9
AZT_3TV_EFV	11
d4T_3TC_EFV	222
d4T_3TC_LPVr	18
d4T_3TC_NVP	22
Average plasma efavirenz concentration, μg/mL (range)	4.64 (0.6–22)

### Comparison of allele frequencies among world populations

The genotypes for all the SNPs were observed in the HIV/AIDS patients, except the *ABCB1* *61G/G* (*rs9282564*) genotype. All *ABCB1* SNPs were in Hardy–Weinberg Equilibrium (HWE). The *ABCB1 61A/G* (*rs9282564*), *3435T/T* (*rs1045642*), *4036G/G* (*rs3842*), *1236T/T* (*rs1128503*), *2677T/A*, and *2677G/A* (*rs2032582*) genotypes were present at frequencies of 0.006, 0.024, 0.036, 0.015, 0.004, and 0.004, respectively, among the South Africans. No individuals with an *ABCB1 3435A* allele was observed in the South African cohort (Table 2), and this is similar to what was reported by Dandara et al. (2011). The allele frequencies of SNPs in the South Africans were compared to the allele frequencies reported previously in other populations (Table 2), available from previous studies or the HapMap project (http://hapmap.ncbi.nlm.nih.gov/).

**Table 2:** **Allele frequencies in the South Africans compared to other populations**.

Population	Reference	*N*	61G	1236T	3435T	2677T	2677A	4036G
Black South Africans^#^	(Dandara et al., 2011)/This study	979	0.003	0.090	0.120	0.040	0.004	0.202
Sotho/Tswana South Africans	This study	127	0.000	0.106	0.110	0.012	0.004	0.165
Xhosa South Africans	This study	107	0.014	0.178*	0.210*	0.098*	0.005	0.224
Zulu South Africans	This study	139	0.000	0.119	0.140	0.022	0.000	0.209
Malawi	Brown et al. (2011)	30	N/A	N/A	0.210	0.000	0.000	N/A
Yoruba	Hapmap	226	0.000	0.124	0.111	0.000*	0.000	0.142
Luhya	Ikediobi et al. (2011)	89	N/A	0.110	N/A	N/A	N/A	N/A
Maasai	Ikediobi et al. (2011)	143	N/A	0.140	0.840*	N/A	N/A	N/A
African-American	Hapmap	46	0.000	0.136	0.071	0.077	0.000	0.000*
Caucasian	Hapmap	226	0.100*	0.451*	0.571*	0.340*	0.042*	0.142
Gujarati Indian	Hapmap	176	0.017	0.597*	0.597*	0.653*	0.000	0.163
Mexican	Hapmap	96	0.052*	0.460*	0.460*	0.430*	0.000	0.230
Toscan	Hapmap	176	0.062*	0.426*	0.466*	0.438*	0.000	0.138
Chinese	Ikediobi et al. (2011)	45	0.000	0.680*	0.580*	N/A	N/A	N/A
Japanese	Hapmap	90	0.000	0.587*	0.459*	0.552*	0.000	0.320*

N/A, not available, *statistically significant difference from the frequencies among the Black South African group^#^.

### Correlation of genetic variation with plasma efavirenz concentration

**Figure 1:**
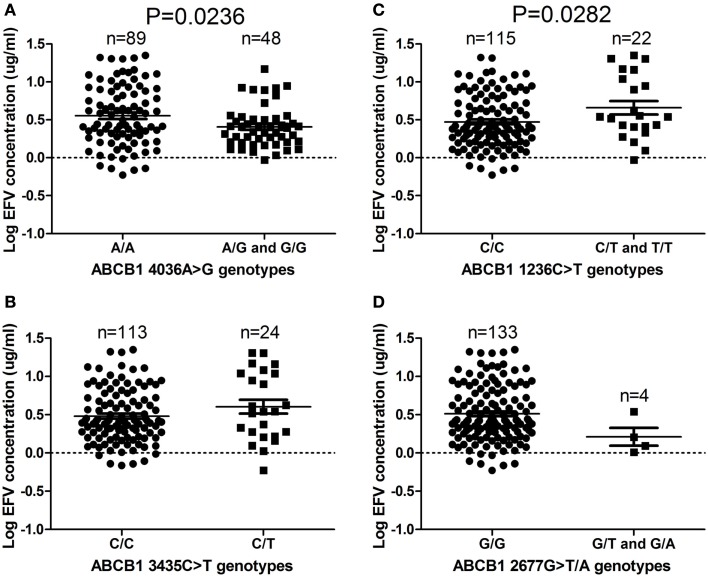
**(A–D)** *ABCB1 4036A/G* and G/G genotypes (*P* = 0.0236) are associated with reduced efavirenz levels, while *1236C/T* and T/T genotypes (*P* = 0.0282) are associated with increased efavirenz levels in South African HIV/AIDS patients. Only significant *P*-values (without Bonferroni correction), using the dominant genetic model, are shown.

The *ABCB1 4036A/G* and *4036G/G* genotypes were associated with significantly decreased efavirenz levels (*P* = 0.0236), compared to the *4036A/A* genotype (Figure 1A). Fewer individuals with the *ABCB1 4036G/G* genotype changed treatment compared to the individuals with the *4036A/A* genotype (Table 3). The *ABCB1 1236C/T* and *1236T/T* genotypes were associated with significantly higher plasma efavirenz concentrations, compared to the *1236C/C* genotype (*P* = 0.0282; Figure 1C). Compared to the *ABCB1 1236C/C* genotype, more individuals with the *1236T/T* genotype changed antiretroviral regimens 1 year post treatment initiation (Table 3). No difference was observed when comparing individuals with efavirenz concentration above 4 μg/mL to those with concentrations below 4 μg/mL, with respect to change in treatment regimens (*P* = 0.571). No significant differences were observed in efavirenz concentrations between the *ABCB1 2677G*>*T/A* and *ABCB1 3435C*>*T* genotypes (Figures 1B,D).

**Table 3:** Frequency of HIV/AIDS patients changing ART regimens within 3 months, 6 months and 1 year post-initiation of treatment*.

Genotype	Treatment initiation (*n*)	3 months	*P*-value	6 months	*P*-value	1 year	*P*-value
**EFV-CONTAINING ARV REGIMEN**							
3TC_TDF_EFV	9	0.11		0.00		0.11	
AZT_3TC_EFV	11	0.00	0.182	0.09	0.993	0.18	0.898
d4T_3TC_EFV	222	0.03		0.05		0.14	
1236C/C	192	0.04		0.05		0.12	
1236C/T	44	0.02	0.399	0.04	0.636	0.18	0.089
1236T/T	3	0.00		0.00		0.50	
3435C/C	192	0.04		0.05		0.13	
3435C/T	44	0.00	0.387	0.05	0.962	0.16	0.653
3435T/T	3	0.33		0.00		0.00	
2677G/G	229	0.03		0.06		0.13	
2677G/T	7	0.00	0.563	0.11	0.666	0.00	0.706
2677T/A and G/A	2	0.00		0.00		0.50	
4036A/A	153	0.03		0.05		0.14	
4036A/G	78	0.03	0.987	0.06	0.834	0.14	0.852
4036G/G	8	0.00		0.00		0.11	

*Only 282 patients had information on treatment regimens. *ABCB1 61A*>*G* was excluded based on being monomorphic.

### Haplotype analysis

**Figure 2:**
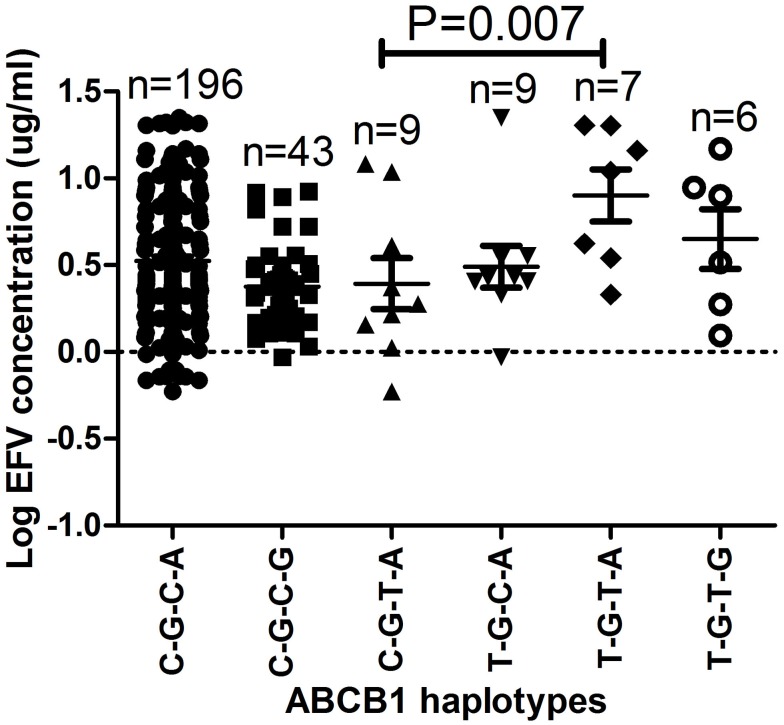
**The effect of *ABCB1* haplotypes on plasma efavirenz concentrations**. Only significant *P*-values after Bonferroni’s correction for multiple testing at significance *P* < 0.01 are shown. The *ABCB1 C-A-C-A*, *C-T-C-A*, and *T-T-T-G* haplotypes were only observed once and thus excluded from the analysis.

Haplotype and efavirenz plasma levels for each patient are presented in supplementary Table A1. The haplotypes with respect to *1236C*>*T*, *2677G*>*T/A*, *3435C*>*T*, and *4036A*>*G* SNPs *C-G-C-A*, *C-G-C-G*, *C-G-T-G*, *T-G-C-A*, *T-G-T-A*, *T-G-T-G*, *T-T-T-A*, and *T-T-T-G* had the following frequencies in the HIV/AIDS patients; 0.67, 0.17, 0.04, 0.03, 0.06, 0.01, 0.001, and 0.01, respectively. The *ABCB1 T-G-T-A* haplotype had the highest mean plasma log_10_ efavirenz concentrations (0.90 μg/mL) compared to 0.49 and 0.65 μg/mL among patients with the *T-G-C-A* or *T-G-T-G* haplotypes, respectively (Figure 2). The efavirenz concentrations differed significantly between individuals with the *ABCB1 C-G-C-G* and *T-G-T-A* haplotypes (*P* = 0.007) and were still significant after Bonferroni’s correction for multiple testing (cut-off significant *P* < 0.01).

### Univariate and multivariate regression analysis of efavirenz concentration

Univariate regression analysis was performed to determine the effect of age, gender, tobacco smoking, alcohol use, BMI at baseline, CD-4 cell count, log_10_ HIV-RNA levels, *ABCB1* haplotypes, *ABCB1 1236C*>*T, 4036A*>*G*, *3435C*>*T*, and *2677G*>*T/A* genotypes on plasma efavirenz concentrations (Table 4). The genotypes of the four SNPs *1236C*>*T*, *2677G*>*T/A*, *3435C*>*T*, and *4036A*>*G* significantly predicted efavirenz concentration individually and were, thus, included in the multivariate analysis together with age, gender, tobacco smoking, alcohol use and BMI at baseline. Stepwise backward regression analysis was then performed to identify the minimum set of independent variables that are predictive of plasma efavirenz levels and to determine the relative contribution of each variable to efavirenz concentration variability. Only three independent variables remained in the final model with *P* < 0.05, including *ABCB1 1236C*>*T* (standardized regression coefficient = 0.24; *P* = 0.004), *ABCB1 4036A*>*G* (standardized regression coefficient = 0.17; *P* = 0.009), and *ABCB1 2677G*>*T/A* (standardized regression coefficient = 0.36; *P* = 0.047). The adjusted coefficient of determination (*R*^2^) for the regression was 0.16, indicating that 16% of the total variance in efavirenz concentrations was explained by the model. *ABCB1 1236C*>*T, 4036A*>*G*, and *2677G*>*T/A* genotypes accounted for 29, 23, and 17% (respectively) of the total variance in plasma efavirenz concentrations. When repeating the multivariate analysis among female patients only, the adjusted coefficient of determination (*R*^2^) for the regression was 0.18, indicating that 18% of the total variance in efavirenz concentrations was explained by the model compared to the 16% explained when males and females were combined. However, the majority of HIV/AIDS patients in our study were female. Further statistical analysis comparing only the genetic- and non-genetic factors in the multivariate analysis, showed that the genetic factors alone explained 11% of variance in efavirenz levels, while the non-genetic factors only explained 3% compared to the 16% explained by the combined multivariate analysis.

**Table 4:** **Univariate and multivariate regression analysis of efavirenz concentration**.

Independent variable	Log_10_ efavirenz, % (95%CI)	*P*	Contribution in model (%)
**UNIVARIATE**			
Age	0.01 (−0.06 to 0.08)	0.738	1.36
Gender	−7.34 (−21.5 to 6.81)	0.307	0.12
Tobacco smoking	−0.75 (−27.2 to 25.7)	0.956	2.60
Alcohol use	−12.4 (−34.3 to 9.45)	0.263	11.9
BMI at baseline	−0.70 (−2.16 to 0.76)	0.344	15.2
CD-4 cell count at baseline	0.01 (−0.06 to 0.07)	0.793	–
CD-4 cell count at 6 months	−0.04 (−0.09 to 0.02)	0.186	–
Log_10_ HIV-RNA at baseline	−11.9 (−21.3 to −2.44)	0.015	–
Log_10_ HIV-RNA at 6 months	10.4 (−7.26 to 28.0)	0.245	–
Genotype (dominant model)			
WT/WT	Ref		
ABCB1 1236C>T	18.6 (2.02 to 35.2)	0.028	28.9
ABCB1 4036A>G	−14.8 (−27.5 to −2.01)	0.024	23.2
ABCB1 3435C>T	12.3 (−3.90 to 28.4)	0.136	0.12
ABCB1 2677G>T/A	−30.0 (−66.4 to 6.47)	0.106	16.6
ABCB1 haplotypes	0.19 (−1.98 to 2.37)	0.862	–
**MULTIVARIATE^#^**			
ABCB1 1236C>T	24.2 (7.81 to 40.6)	0.004	–
ABCB1 4036A>G	−16.6 (−29.1 to −4.15)	0.009	–
ABCB1 2677G>T/A	−35.9 (−71.3 to −0.43)	0.047	–

*^#^Only significant covariates in the multivariate regression analysis are shown*.

### Correlation of genetic variation with clinical parameters

**Figure 3:**
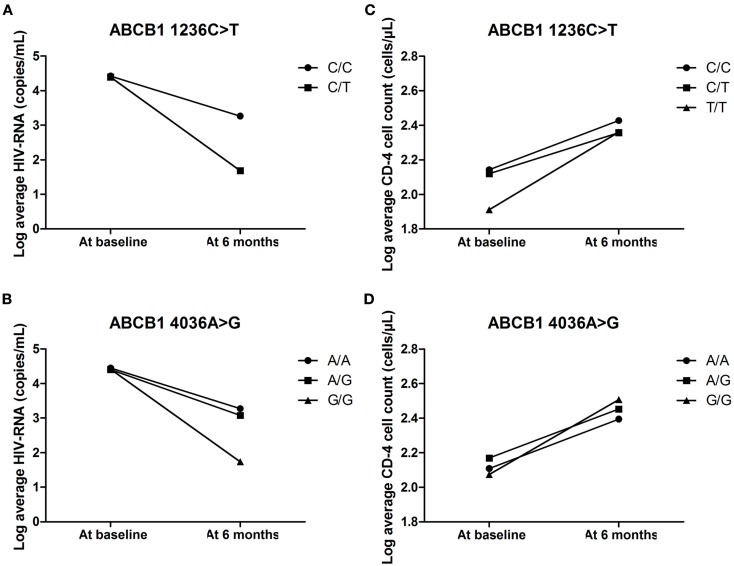
**(A–D)** The effect of *ABCB1 4036A*>*G* and *1236C*>*T* on average HIV-RNA and CD-4 cell count at baseline and 6 months post-initiation of treatment.

The average CD-4 cell count and viral load of individuals with the different genotypes for *ABCB1 1236C*>*T* and *4036A*>*G* were compared at baseline and also 6 months post-initiation of ARV therapy. The *ABCB1 1236C/T* and *4036G/G* genotypes were associated with the biggest decreases in viral load at 6 months (as shown in Figures 3A,B). None of the individuals with the *ABCB1 1236T/T* genotype had information on viral load at baseline or 6 months post-initiation of treatment. The *ABCB1 1236T* allele is associated with decreased expression of P-gp (Gow et al., 2008) and the *ABCB1 4036G* allele is associated with increased expression of P-gp. However, there were no major genotype associated differences in the recovery of the CD-4 cells with all genotypes showing a positive response (Figures 3C,D).

## Discussion

### Heterogeneity of african populations in terms of genetics

To our knowledge, the present study is the first to report on the allele and genotype frequencies of the *ABCB1 61A*>*G, 2677G*>*T/A*, and *4036A*>*G* polymorphisms in the South African population. However, the other SNPs have been previously reported by Dandara et al. (2011). This data contributes to the accumulation of information on genetic variants of pharmacogenetics relevance among Africans. The allele frequencies of the genetic polymorphisms in South Africans were also compared to the frequencies among other African, African-American, Asian, and Caucasian populations (Table 2). As expected there are significant differences in the allele frequencies between African populations and Caucasians, for example, the allele frequency of the *ABCB1 3435T* allele in African populations ranges from 0.07 to 0.12, but is present at a frequency of almost 0.6 in Caucasian individuals. Differences in allele frequencies between the South African population compared with other African populations were also observed. The allele frequencies of the *ABCB1 4036G*, *2677T*, and *2677A* alleles were different to the frequencies reported in the Yoruba individuals (*P* < 0.0001). The differences in allele frequencies between the African and Caucasian individuals show that therapeutic drugs, including efavirenz, may not have similar effectiveness in different populations when given at standard dosages. Similarly, fine scale genetic structure exists within the African population which, therefore, should not be treated as one population.

### Implications for disease or drug treatment and possible development of diagnostic tools

We observed lower plasma efavirenz concentrations among individuals with the *ABCB1 4036A/G* and *4036G/G* genotypes and this could perhaps be as a result of the disruption of a miRNA binding site in the 3′UTR of *ABCB1*. Five poorly conserved miRNAs namely; miR-129, miR-491, miR-4795, miR-561, and miR-4717 have been predicted to target the 3′UTR region surrounding the *ABCB1 4036A*>*G* SNP using the TargetScanHuman 6.1 miRNA target prediction software. Disruption of these sites could potentially cause reduced transport of efavirenz by *ABCB1* resulting in lower plasma efavirenz levels. In a different study among Ugandans, the *ABCB1 4036A/G* and *4036G/G* genotypes were associated with higher efavirenz bioavailability (Mukonzo et al., 2009). In the current study, *ABCB1 1236C/T* and *1236T/T* genotypes were associated with high plasma efavirenz concentrations, but there are conflicting reports on the effect of *ABCB1 1236C*>*T* genotypes in tacrolimus, cyclosporine, and sirolimus drug responses (Kuzuya et al., 2003; Anglicheau et al., 2004; Haufroid et al., 2004), making it difficult to draw conclusions. There are conflicting reports as well for the role or effects of *ABCB1 2677G*>*T/A* and *3435C*>*T* polymorphisms (Schwab et al., 2003; Leschziner et al., 2007). Haas et al. (2005) reported an association between the *ABCB1 3435T/T* genotype and a decreased likelihood of virologic failure and decreased resistance to efavirenz, but not with plasma efavirenz exposure.

Clinical parameters such as CD-4 cell count, viral load, disease stage, hemoglobin, AST, and ALT levels were used as indicators of ARV treatment efficacy, underlying liver disease and disease progression in the HIV/AIDS patients. As expected, efavirenz-containing HAART led to a general (48%) increase in CD-4 cell count (cells/μL) and a 94% decrease in viral load (copies/mL) when baseline levels where compared to levels at 6 months post-initiation of treatment. Failure of reduction in viral load and emergence of opportunistic infections after 6 months led to ARV switching. Sustained viral load after 6 months and the presence of opportunistic infections are indications of possible treatment failure or non-adherence. On the other hand, other studies have shown that high plasma efavirenz concentrations are associated with development of adverse drug events leading to drug discontinuation (Marzolini et al., 2001; Lubomirov et al., 2011; Wyen et al., 2011).

## Conclusion

The current study showed that the drug transporter *ABCB1* contributes in predicting response to efavirenz treatment in the South African HIV/AIDS population. The *ABCB1 4036A*>*G* and *1236C*>*T* polymorphisms were significantly correlated with low and high plasma efavirenz concentration levels, respectively. However, this data should be taken together with the variation in *CYP2B6* which has a profound effect on efavirenz metabolism. The *CYP2B6 516G*>*T* SNP is known to be associated with high plasma efavirenz levels and the combined effect of *CYP2B6* together with *ABCB1* SNPs will be more informative in predicting response to efavirenz treatment. This strongly supports the development of a pharmacogenetic suite of gene variants to assist in deciding the HAART regimen for HIV/AIDS treatment in a clinical setting as well as the starting ARV dosage.

## Materials and Methods

### Research participants

All participants provided written informed consent and study approval was obtained from the University of Cape Town Health Science Faculty Research Ethics Committee, Cape Town, South Africa and the University of Witwatersrand Human Research Ethics Committee, Gauteng, South Africa. The research was performed in accordance with the guidelines of the Helsinki Declaration of 2008. Two hundred and eighty-two (*n* = 282) South African HIV/AIDS patients receiving efavirenz-based treatment for at least 6 months, were recruited to participate in this study. All subjects were of Bantu origin and comprised of Sotho/Tswana from Gauteng and Xhosa subjects from the Western Cape Province, South Africa. All subjects gave information on their ethnicity, age, health status (including self-reported adherence to treatment or pill counts), dietary, and smoking habits.

A 5 mL whole blood sample was obtained from each subject, and used for plasma sample collection as well as DNA extraction. DNA was isolated using a salting-out method adapted from Gustafson et al. (1987) or the GenElute™ Blood Genomic DNA Kit (Sigma-Aldrich, St. Louis, MO, USA). Steady-state plasma samples were available for 137 HIV/AIDS patients 12–16 h post dose with efavirenz. Plasma efavirenz concentrations were determined by LC/MS/MS (API 4000 triple quadrupole MS/MS Applied Biosystems, South Africa) according to the method by Chi et al. (2002).

### Selection of SNPs and genotyping methods used

Five previously reported SNPs in *ABCB1* [GenBank accession: NM_000927.4], namely *61A*>*G, 1236C*>*T, 2677G*>*T/A*, *3435C*>*T*, and *4036A*>*G* were selected for investigation based on having minor allele frequencies above 10% in the African-Americans, South African, or other African populations and were genotyped using SNaPshot mini-sequencing (Table 5).

**Table 5:** **PCR and SNaPshot amplification conditions for *ABCB1* SNPs (GenBank accession: NM_000927.4)**.

SNP	Primer sequence (5′-3′)	Ta (°C)	PCR product	References
4036A>G	F: CCTCAGTCAAGTTCAGAGTCTTCA	54°C	297	Designed
	R: TCACAGGCAGTTGGACAAG			
	SNaPshot primer: TCTTGGCAGAAACTGCAAAAGGAGATTGAT			
3435C>T	F: ACTCTTGTTTTCAGCTGCTTG	54°C	230	Rhodes et al. (2007)
	R: AGAGACTTACATTAGGCAGTGACTC			
	SNaPshot primer: ACTCGTCCTGGTAGATCTTGAAGGG			
2677G>T/A	F: ATGGTTGGCAACTAACACTGTTA	54°C	206	Rhodes et al. (2007)
	R: AGCAGTAGGGAGTAACAAAATAACA			
	SNaPshot primer: CTTCGACCTAAGTGGAGAATGAGTTATTCTAAGGA			
1236C>T	F: TGTGTCTGTGAATTGCCTTGAAG	51°C	228	Rhodes et al. (2007)
	R: CCTCTGCATCAGCTGGACTGT			
	SNaPshot primer: TTAATTAATCAATCATATTTAGTTTGACTCACCTTCCCAG			
61A>G	F: CTGCGGTTTCTCTTCAGGTC	51°C	149	Designed
	R: GATTCCAAAGGCTAGCTTGC			
	SNaPshot primer: CTCCTTTGCTGCCCTCAC			

Each PCR reaction contained, 50 ng of genomic DNA, 1X Green GoTaq Flexi Reaction Buffer (Promega Corporation, Madison, WI, USA), 0.2 mM of each of the deoxynucleotide triphosphates (dNTPs; Bioline, London, UK); 1.5 mM MgCl_2_ (Promega Corporation, Madison, WI, USA); 40 pmol of the forward and reverse primers (Integrated DNA Technologies, Inc., Coralville, IA, USA); 1 U of GoTaq Flexi DNA Polymerase (Promega Corporation, Madison, WI, USA). The PCR reactions were carried out using a “MyCycler Thermal cycler” from Bio-Rad. PCR conditions were as follows: 3 min at 94°C; 40 cycles of 94°C for 30 s, the annealing temperature specific to each SNP for 30 s, 72°C for 50 s; and 10 min at 72°C for final extension.

Five microliters of each PCR product was pooled and 10 μL of the combined PCR products were cleaned using 1.5 U shrimp alkaline phosphatase (Fermentas Life Sciences, Burlington, Canada) and 2 U *ExoI* (Fermentas Life Sciences, Burlington, Canada) in a total reaction volume of 20 μL. The shrimp alkaline phosphatase and *ExoI* reaction was incubated at 37°C for 1 h and the enzymes were inactivated at 75°C for 15 min. SNaPshot single base extension of the genetic polymorphisms was performed on the “GeneAmp^®^ PCR System 9700 version 3.08′′ (Applied Biosystems, Carlsbad, CA, USA) using the SNaPshot cycling programme as 96°C for 10 s, and then repeated for 25 cycles at 50°C for 5 s and 60°C for 30 s. The SNaPshot reaction (10 μL) contained 1 μL ABI Prism^®^ SNaPshot™ Multiplex Kit (Applied Biosystems, CA, USA) and the pooled internal SNaPshot primers (Integrated DNA Technologies, Inc., Coralville, IA, USA). Following the SNaPshot reaction, the clean-up reaction was repeated using 1 U shrimp alkaline phosphatase using cycling conditions as mentioned before, and capillary electrophoresis was performed using a ABI 3130xl Genetic Analyzer (Applied Biosystems, Carlsbad, CA, USA). SNaPshot results were analyzed on the GeneMapper © Software version 4.1 (Applied Biosystems, Carlsbad, CA, USA).

### Statistical analysis

Statistical analysis was performed using the Graphpad Prism (Version 5, GraphPad Software Inc., San Diego, CA) and STATA (Version 11, StatSoft, USA) statistical programs. *ABCB1* haplotypes were inferred using Phase v2.1 (Stephens et al., 2001; Stephens and Donnelly, 2003; Stephens and Scheet, 2005). Pearson’s χ^2^-test and Fisher’s exact test were used to compare the allele frequencies to results previously published in populations of different ethnicity. Fisher’s exact test was also used to compare change in treatment regimen between the *ABCB1* genotypes. One-way analysis of variance, followed by Bonferroni’s multiple comparison tests, was used to determine the effect of *ABCB1* haplotypes on plasma log_10_ efavirenz levels. Genotypes were dichotomized according to the dominant genetic model (wildtype = 0 and heterozygote/homozygote variants = 1). Univariate regression analysis was applied to log_10_ efavirenz concentrations as dependant variable and the percentage change in efavirenz levels, with the 95% CI, was calculated as 100 × regression coefficient. Multivariate regression analysis was performed by including covariates from the univariate analysis, followed by stepwise backward removal. TargetScanHuman 6.1 miRNA target prediction software were used to predict miRNA binding to the *ABCB1 3′UTR*. The following equation was used to calculate the sample size required to achieve a 99% confidence-interval: *N* = [DEFF * Np(1−*p*)]/[(d2/Z21−α/2 * (*N*−1) + *p* * (1−*p*)]. DEFF is defined as the design effect, *Z* is the value for 99% confidence, *d* is an α-value = 0.05, while *p* is the frequency of the variant allele. A DEFF-value of 1 was used for random sampling, a *Z*-value of 1.96 and allele frequency of 0.1 was used to calculate the sample size of *N* = 239 samples. All statistical tests were performed two tailed, and statistical significance was defined as *P* < 0.05.

## Conflict of Interest Statement

The authors declare that the research was conducted in the absence of any commercial or financial relationships that could be construed as a potential conflict of interest.

## Appendix

**Table A1:** ***ABCB1* haplotype composition for each HIV/AIDS patient where steady-state plasma efavirenz levels were available**.

HIV/AIDS patients	ABCB1 haplotype combination*	Log_10_ efavirenz levels (μg/mL)
1	C-G-C-A/C-G-C-A	0.111
2	C-G-C-A/C-G-C-A	0.838
3	C-G-C-A/C-G-T-A	0.023
4	C-G-C-A/C-G-C-A	−0.013
5	C-G-C-A/C-G-C-A	0.676
6	C-G-C-A/C-G-C-G	0.507
7	C-G-C-A/C-G-T-A	0.275
8	C-G-C-A/C-G-C-G	0.199
9	C-G-C-A/C-G-C-G	0.428
10	C-G-C-A/C-G-C-G	0.327
11	C-G-C-A/C-G-T-A	−0.229
12	C-G-C-A/C-G-C-G	0.917
13	C-G-C-A/C-G-C-G	0.208
14	C-G-C-A/C-G-C-G	0.415
15	C-G-C-A/C-G-C-G	0.478
16	C-G-C-A/C-G-C-A	0.321
17	C-G-C-A/C-G-C-G	0.276
18	C-G-C-A/T-G-T-A	0.624
19	C-G-C-A/C-G-T-A	1.083
20	C-G-C-A/C-G-C-G	0.170
21	C-G-C-A/C-G-C-G	0.445
22	C-G-C-A/C-G-T-A	0.611
23	C-G-C-A/C-G-C-G	0.419
24	C-G-C-A/C-G-C-G	0.558
25	C-G-C-A/C-G-C-A	0.622
26	C-G-C-A/C-G-C-A	0.267
27	C-G-C-G/C-G-C-G	0.107
28	C-G-C-A/T-G-T-G	1.170
29	C-G-C-A/C-G-C-A	0.083
30	C-G-C-A/C-G-C-A	0.390
31	C-G-C-A/C-G-C-A	0.408
32	C-G-C-A/C-G-C-A	0.720
33	C-G-C-A/C-G-C-A	0.378
34	C-G-C-A/C-G-T-A	0.157
35	C-G-C-A/C-G-C-G	0.172
36	C-G-C-A/C-G-C-A	0.029
37	C-G-C-A/C-G-C-A	0.902
38	C-G-C-A/C-G-C-A	0.380
39	C-G-C-A/C-G-C-A	1.016
40	C-G-C-A/C-G-C-A	0.880
41	C-G-C-A/T-G-C-A	0.407
42	C-G-C-A/C-G-C-G	0.819
43	C-G-C-A/C-G-C-A	0.780
44	C-G-C-A/C-G-C-A	1.316
45	C-G-C-A/C-G-C-G	0.112
46	C-G-C-G/T-G-C-A	0.554
47	C-G-C-A/C-G-C-G	0.419
48	C-G-C-A/C-G-C-A	0.352
49	C-G-C-A/C-G-C-A	1.141
50	C-G-C-A/T-T-T-G	0.539
51	C-G-C-A/T-G-T-A	1.303
52	C-G-C-A/C-G-C-A	0.927
53	C-G-C-A/C-G-C-G	0.298
54	C-G-C-A/C-G-C-G	0.892
55	C-G-C-A/C-G-C-A	0.422
56	C-G-C-A/T-G-T-G	0.899
57	C-G-C-A/T-G-C-A	0.428
58	C-G-C-G/C-G-C-G	0.723
59	C-G-C-A/T-T-T-G	0.205
60	C-G-C-A/C-G-C-A	0.353
61	C-G-C-A/C-G-C-G	0.252
62	C-G-C-G/C-G-C-G	0.499
63	C-G-C-A/C-G-C-A	0.442
64	C-G-C-A/T-G-T-G	0.095
65	C-G-C-A/C-G-C-A	0.649
66	C-G-C-G/T-G-C-A	0.405
67	C-G-C-A/C-G-C-A	0.495
68	C-G-C-A/C-G-C-G	0.413
69	C-G-C-A/C-G-C-A	0.619
70	C-G-C-A/C-G-C-A	0.196
71	C-G-C-A/T-G-T-A	1.306
72	C-G-C-A/C-G-C-G	0.328
73	C-G-C-A/C-G-C-A	0.932
74	C-G-C-A/C-G-C-A	0.315
75	C-G-C-A/C-G-C-A	1.322
76	C-G-C-A/C-G-C-A	0.585
77	C-G-C-A/C-G-C-A	0.623
78	C-G-C-G/C-G-T-A	0.215
79	C-G-C-A/C-G-C-A	1.125
80	C-G-C-A/C-G-C-A	0.294
81	C-G-C-A/T-G-T-G	0.947
82	C-G-C-A/T-G-T-A	1.160
83	C-G-C-A/C-G-C-A	0.989
84	C-G-C-A/C-G-C-A	0.072
85	C-G-C-A/C-G-C-A	0.415
86	C-G-C-A/C-G-C-A	0.214
87	C-G-C-A/C-G-C-A	0.874
88	C-G-C-A/C-G-C-G	0.450
89	C-G-C-A/C-G-C-A	−0.140
90	C-G-C-A/C-G-T-A	1.037
91	C-G-C-A/C-G-C-A	0.381
92	C-G-C-A/C-G-C-A	−0.143
93	C-G-C-A/C-G-C-G	0.076
94	C-G-C-A/C-G-C-G	0.924
95	C-G-C-A/C-G-C-G	0.033
96	C-G-C-A/C-G-C-A	0.946
97	C-G-C-A/C-G-C-A	−0.164
98	C-G-C-G/T-G-C-A	−0.029
99	C-G-C-A/C-G-C-A	−0.105
100	C-G-C-A/C-G-C-A	0.645
101	C-G-C-A/C-G-C-A	0.911
102	C-G-C-A/C-G-C-G	0.394
103	C-G-C-A/T-G-T-G	0.274
104	C-G-C-A/C-G-C-G	0.313
105	C-G-C-A/C-G-C-G	0.164
106	C-G-C-A/C-G-T-A	0.371
107	C-G-C-A/C-G-C-A	0.264
108	C-G-C-A/C-T-C-A	0.010
109	C-G-C-A/C-G-C-G	0.111
110	C-G-C-A/C-G-C-A	1.109
111	C-G-C-A/C-G-C-A	0.671
112	C-G-C-A/C-G-C-A	0.324
113	C-G-C-A/C-G-C-A	0.431
114	C-G-C-A/T-G-C-A	0.431
115	C-G-C-A/C-G-C-A	0.566
116	C-G-C-A/C-G-C-A	0.780
117	C-G-C-A/T-G-T-G	0.516
118	C-G-C-A/C-G-C-G	0.276
119	T-G-C-A/T-G-T-A	0.541
120	C-G-C-A/C-G-C-A	0.821
121	C-G-C-A/T-G-T-A	1.038
122	C-G-C-A/C-G-C-A	0.364
123	C-G-C-A/C-G-C-A	1.106
124	C-G-C-A/C-G-C-A	0.584
125	C-G-C-A/C-G-C-A	1.093
126	C-G-C-A/C-G-C-A	0.692
127	C-G-C-A/C-G-C-G	0.340
128	C-G-C-A/C-G-C-A	0.751
129	C-G-C-A/C-G-C-A	0.192
130	C-A-C-A/C-G-C-A	0.092
131	C-G-C-A/C-G-C-A	0.192
132	C-G-C-A/C-G-C-A	0.106
133	C-G-C-A/C-G-C-A	0.941
134	C-G-C-A/C-G-C-A	0.204
135	C-G-C-A/T-G-C-A	1.348
136	T-G-C-A/T-G-T-A	0.329
137	C-G-C-A/C-G-C-G	0.271

***ABCB1* haplotypes with respect to *1236C*>*T-2677G*>*T/A-3435C*>*T-4036A*>*G*, *61A*>*G* was excluded from the haplotype based on being monomorphic*.
